# A two-item screening of maternal or infant perceived life threat during childbirth prospectively associated with childbirth-related posttraumatic stress symptoms up to six months postpartum: two observational longitudinal studies

**DOI:** 10.3389/fpsyt.2024.1360189

**Published:** 2024-04-09

**Authors:** Leah Gilbert, Vania Sandoz, Camille Deforges, Antje Horsch

**Affiliations:** ^1^ Department Woman-mother-child, Lausanne University Hospital, Lausanne, Switzerland; ^2^ Charles Perkins Center, Nepean Clinical School, Faculty of Medicine and Health, University of Sydney, Penrith, NSW, Australia; ^3^ Child Abuse and Neglect Team, Department Woman-mother-child, Lausanne University Hospital, Lausanne, Switzerland; ^4^ Institute of Higher Education and Research in Healthcare, University of Lausanne, Lausanne, Switzerland

**Keywords:** birth trauma, perceived infant threat, perceived maternal threat, threat perception, emergency caesarean section, childbirth, CB-PTSD

## Abstract

**Objective:**

This study investigated prospective relationships between the perception of threat to one’s own life or to that of one’s infant during childbirth and maternal childbirth-related posttraumatic stress symptoms (CB-PTSS) and probable childbirth-related posttraumatic stress disorder (CB-PTSD) in a community and a community and an emergency cesarean section (ECS) sample.

**Methods:**

Study samples included 72 mothers from a community sample and 75 mothers after emergency cesarean section. Perceived maternal and infant life threat were assessed at ≤1 week postpartum. Maternal CB-PTSS and probable CB-PTSD were assessed with validated questionnaires up to 6 months postpartum. Covariates were extracted from hospital records. Secondary data analysis with logistic and linear regressions was performed.

**Results:**

Globally, mothers were significantly more likely to perceive their infant’s life to be threatened, rather than their own. Both types of perceived threat were prospectively but differentially associated with maternal CB-PTSS and probable CB-PTSD at 4-6 weeks and 6 months postpartum. Statistical significance was set at p<0.05.

**Conclusion:**

The type of perceived threat differently influences maternal CB-PTSS and probable CB-PTSD up to 6 months postpartum. These results may be the basis for the development of a short screening instrument after traumatic childbirth in clinical settings. Future studies need to assess the psychometric properties and acceptability of such a brief screening tool.

## 1 Introduction

According to the posttraumatic stress disorder (PTSD) criterion A of the Diagnostic and Statistical Manual of Mental Disorders fifth edition (DSM-5), a traumatic event is a direct or indirect exposure to a life threat or severe injury (1). As parents can perceive a maternal and/or infant life threat during childbirth, this event can fulfil the PTSD criterion A and lead to childbirth-related PTSD (CB-PTSD) (2–4). Up to 33-46% of mothers appraise their childbirth as traumatic (5–8).

CB-PTSD has four symptom clusters: intrusions (e.g., childbirth-related flashbacks and nightmares); avoidance of birth-related cues (e.g., not talking about the birth); negative cognitions and mood (e.g., anhedonia and low mood); and hyperarousal (e.g., increased startle response and hypervigilance towards the infant) (2, 9). Childbirth-related posttraumatic stress disorders has been shown to consist of birth-related and general posttraumatic stress symptoms (10), and researchers have recently argued that CB-PTSD is a distinct sub-type of PTSD (3). Meta-analyses concluded that 3-4% of mothers meet CB-PTSD diagnostic criteria in community samples whereas, in high-risk populations (e.g., emergency caesarean section (ECS)), this increases to 16-19% (11, 12). However, the prevalence of sub-threshold childbirth-related posttraumatic stress symptoms (CB-PTSS) is much higher (12.3% and 21.1%) (13). Evidence showed associations between CB-PTSS and adverse family outcomes (14–16) (17–19).

Given the significant consequences of CB-PTSS on family outcomes (such as breastfeeding, parent-infant bonding, infant development, couple relationship satisfaction) and its economic costs due to increased healthcare utilization (20), early identification of women at risk of CB-PTSS is crucial (12, 21). A screening procedure suitable to the maternity context could facilitate the implementation of early preventive strategies for mothers who otherwise may avoid care in the postpartum period for themselves or their infant. The subjective birth appraisal is a determinant as it is one of the strongest predictors of a traumatic childbirth experience (19, 22–24). An apparently non-traumatic childbirth, as described by healthcare professionals, can be experienced as traumatic by the mother, and vice-versa (19, 22, 25, 26). Therefore, at-risk mothers cannot be identified only based on medical criteria. To date, no validated tool exists to assess maternal risk of developing CB-PTSS shortly after childbirth. Such an acceptable, early screening tool would represent an important clinical opportunity (27).

During childbirth, both lives of mothers and their infants can be threatened, and maternal perceived life threat can contribute to the development of CB-PTSS. While the literature is consistent regarding the predictive nature of maternal perceived life threat perception for oneself (7, 23), the role of a maternal perceived life threat for their infant is less well understood during childbirth. A few studies investigated the role of parental perception of life threat to their child on parental PTSS following other traumatic events (28, 29). For instance, parental perception of the child’s life threat following traffic-related accidents prospectively predicted parental acute stress severity, which was associated with subsequent parental PTSD severity (28). Another longitudinal study with parents of children exposed to a burn observed an impact of parental perceived child’s life threat on intrusion and avoidance symptoms at one-month post-trauma (29). Regarding childbirth, to our knowledge, no study examined the role of maternal perception of infant life threat on maternal CB-PTSS. One experimental cross-sectional study investigated its role on maternal physiological stress reactivity and found that mothers with traumatic childbirth had an altered physiological stress reactivity at three days postpartum, when adjusting for maternal perceived infant life threat during childbirth (30). This supports the assumption of a specific role of the latter in CB-PTSS development. Overall, additional research is needed to better understand the relationship between the perception of threat and CB-PTSS.

This secondary data analysis of two observational longitudinal studies aimed to investigate prospective relationships between the perception of threat, either to one’s own life or to that of one’s infant, and CB-PTSD symptoms at four to six weeks and six months postpartum, in a community sample and an ECS sample. The same associations were explored when applying a cut-off of two or three for the two questions (rated on a Likert scale from 1-7), as the objective was to make the instrument easy to use and interpret for clinicians.

## 2 Methods

### 2.1 Design, study population, and procedure

For this secondary data analysis, data for the community and ECS samples had been collected as part of two clinical research studies in a Swiss University Hospital.

#### 2.1.1 Community sample

Data were derived from the Lausanne Perinatal Wellbeing Cohort, a prospective population-based cohort study (4, 17, 31). Women were recruited during the third trimester of pregnancy. They were included if they were at least 18 years old and understood French, but were excluded if they had a psychotic illness, severe intellectual disability, or, in case of stillbirth. Participants were invited to complete online questionnaires regarding mental health at one week, one, three, and six months postpartum on Sphinx iQ2. Participants who had completed the two screening questions assessing their perception of maternal or infant life threat between January 2013 and August 2021, were included in this study (*n* = 72). All participants gave their written consent. The study was approved by the ethics committee for research in humans of the Canton de Vaud (approval number: 480/2012).

#### 2.1.2 Emergency cesarean section sample

Data were derived from the Swiss TrAumatic biRth Trial (NCT03576586.), see Sandoz et al. for further details (32). Women were approached on the maternity postnatal ward, before six hours postpartum. Women aged over 18 years were eligible to participate if they were traumatized by their ECS at more than 34 weeks of gestation (i.e., met DSM-5-TR criterion A) and had given birth to a live infant. Exclusion criteria were insufficient French skills, hospitalization of the infant, established intellectual disability or psychotic illness, several maternal or infant illnesses, and alcohol abuse or illegal drug use during pregnancy. In the present study, analyses included data only from the control group, and focused on data collected before six hours, at six weeks, and six months postpartum. Included participants (*n* = 75) were recruited between August 2018 and October 2021. The study was approved by the ethics committee for research in humans of the Canton de Vaud (approval number: 2017-02142).

### 2.2 Measures

#### 2.2.1 Maternal perception of threat

Maternal perception of threat was assessed by asking the mothers two questions, i.e., “*To what extent did you perceive your life to be in danger?*” (life threat), “*To what extent did you perceive your infant’s life to be in danger?*” (infant threat), ranging on a Likert scale from 1 = *not at all* to 7 = *extremely*. The development of these two items was based on previous findings and DSM-IV-TR (32, 33). A higher score indicated a higher perceived threat by the mother. Perceived threat was assessed at one week postpartum in the community sample, and within the first six hours postpartum in the ECS sample.

#### 2.2.2 CB-PTSS and probable CB-PTSD

In the community sample, CB-PTSS and probable CB-PTSD were assessed with the French version of the Post-traumatic Diagnostic Scale (PDS-F) (34), a self-report questionnaire, with each of the 17 items corresponding to a PTSD symptom, as described in DSM-IV (35). The PDS-F has four response categories reflecting symptom frequency over the past month, from 0 to 3. A total CB-PTSS severity score can be computed by adding each item score (range: 0–51), with higher total scores indicating more severe CB-PTSS, and a total score ≥ 15 indicating probable CB-PTSD diagnosis. The PDS-F has three symptom cluster subscales: intrusions, avoidance, and arousal. Symptoms are considered as present if the item score is higher than one. CB-PTSS and probable CB-PTSD were measured at one month and six months postpartum. In this study, PDS-F Cronbach alpha was 0.90 at one month and 0.81 at six months.

In the ECS sample, CB-PTSS and probable CB-PTSD were measured at six weeks and six months postpartum, with the French version of the PTSD Checklist for DSM-5-TR (PCL-5) (2, 36). The PCL-5 is a 20-item self-report questionnaire, each item measuring the presence of a PTSD symptom linked to childbirth, over the past month (2), on a five-point scale ranging from 0 to 4. Higher total scores (range: 0−80) reflect more severe symptoms, with a total score ≥ 31 indicating probable CB-PTSD (36). It is also possible to compute a total score with the four PTSD symptom clusters (2) (intrusions, avoidance, negative alteration in cognitions and mood, and hyperarousal). In this study, PCL-5 Cronbach alpha was 0.91 at six weeks and 0.93 at six months.

#### 2.2.3 Medical and sociodemographic information

Medical and sociodemographic information was extracted from the hospital birth records. This included weeks of gestation at birth, Apgar score (37) five minutes after birth, parity, infant’s birth weight (kilograms) and birth modality. Maternal age and education were self-reported at the first study visits for both samples.

### 2.3 Data analysis

For this secondary data analysis, all analyses were carried out with R (38). Descriptive statistics were conducted for socio-demographic variables (**Table 1**). Continuous and normally distributed variables were described as means and standard deviations and ordinal outcomes were described as frequencies and percentages. Statistical significance was set at p<0.05.

**Table 1:** Descriptive characteristics of the community and emergency caesarean section (ECS) samples.

Variables	Community sample *N* = 72		ECS sample *N* = 75	
	*N, m (SD)*	*N (%)*	*N, m (SD)*	*N (%)*
Maternal age	41, 33.61 (4.00)		59, 34.18 (4.48)	
Education				
Primary education		0 (0%)		1 (1.33%)
Secondary education		8 (11.11%)		3 (4.00%)
Apprenticeship/University		31 (43.06%)		48 (64.00%)
Missing		33 (45.83%)		23 (30.67%)
Parity	41, 0.61 (0.89)		60, 0.38 (0.61)	
0		25 (34.72%)		42 (56.00%)
1		12 (16.66%)		15 (20.00%)
2		2 (2.77%)		3 (4.00%)
3		2 (2.77%)		0 (0%)
Missing		31 (43.08%)		15 (20.00%)
Weeks of gestation at birth	41, 39.72 (1.37)		60, 39.55 (1.74)	
Apgar score at five minutes	40, 9.53 (0.85)		59, 9.29 (0.93)	
Birth weight (in kgs)	41, 3.32 (0.49)		60, 3.31 (0.50)	
Birth Modality				
Vaginal		26 (36.11%)		
Forceps		3 (4.17%)		
Vacuum		1 (1.39%)		
Elective C-section		6 (8.33%)		75 (100%)
Emergency C-section		5 (6.94%)		
Missing		31 (43.06%)		
Perceived maternal life threat – six hours	NA		75, 2.63 (1.04)	
Perceived infant life threat – six hours	NA		75, 3.67 (2.12)	
Perceived maternal life threat – one week	72, 1.56 (1.09)		NA	
Perceived infant life threat – one week	72, 2.61 (1.90)		NA	
PDS-F Total Score – one month	53, 5.11 (6.56)		NA	
PDS-F cut-off for PTSD (≥15) – one month		4 (5.56%)		NA
Meeting the criteria for PTSD on the PDS-F – one month		6 (18.34%)		NA
Hyperarousal Score on the PDS-F – one month	53, 2.30 (2.58)		NA	
Avoidance Score on the PDS-F – one month	53, 2.04 (3.38)		NA	
Intrusion Score on the PDS-F – one month	53, 0.77 (1.64)		NA	
PDS-F Total Score – six months	44, 3.32 (3.45)		NA	
PDS-F cut-off for PTSD (≥15) – six months		0 (0%)		NA
Meeting the criteria for PTSD on the PDS-F – six months		2 (2.78%)		NA
Hyperarousal Score on the PDS-F – six months	44, 2.05 (2.11)		NA	
Avoidance Score on the PDS-F – six months	44, 0.84 (1.51)		NA	
Intrusion Score on the PDS-F – six months	44, 0.43 (0.93)		NA	
PCL-5 Total Score – six weeks	NA		57, 9.49 (10.20)	
PCL-5 Cut-off for PTSD (≥31) – six weeks		NA		2 (2.67%)
Reexperiecing on the PCL-5 – six weeks	NA		57, 1.79 (2.91)	
Avoidance on the PCL-5 – six weeks	NA		57, 0.86 (1.60)	
Negative Alterations of cognitions and mood on the PCL-5 – six weeks	NA		57, 3.14 (3.50)	
Arousal on the PCL-5 – six weeks	NA		57, 3.70 (4.00)	
PCL-5 Total Score – six months	NA		49, 9.96 (11.78)	
PCL-5 Cut-off for PTSD (≥31) – six months		NA		5 (6.67%)
Reexperiecing on the PCL-5 – six months	NA		49, 1.65 (2.93)	
Avoidance on the PCL-5 – six months	NA		49, 0.76 (1.79)	
Negative Alterations of cognitions and mood on the PCL-5 – six months	NA		49, 3.84 (4.88)	
Arousal on the PCL-5 – six months	NA		49, 3.71 (4.18)	

NAs represent the variables that were not calculated in the specific populations.

PDS-F: French version of the Post-traumatic Diagnostic Scale (range: 0−51).

PCL-5: Post-Traumatic Stress Disorder Checklist for Diagnostic and Statistical Manual of Mental Disorders, fifth edition (range: 0−80).

PTSD: Post-Traumatic Stress Disorder.

Statistical significance level was defined as p <0.05 and presented in bold.

When investigating the prospective association between *life threat* or *infant threat* as a score (first aim) or as a cut-off (second aim) and CB-PTSS at four to six weeks and six months, we conducted logistic (meeting the cut-off for probable CB-PTSD) and linear (for all other outcomes) regressions.

For all regressions, we used two models. In model 1, we did not adjust the regressions for covariates. In model 2, displayed in the **Supplementary Materials**, we controlled for maternal age and mode of delivery when the predictor was *life threat* and for Apgar score at five minutes and birth weight when it was *infant threat* as these variables are associated to one another according to the literature (19, 22–24). We used one-tailed tests in all our analyses, as we hypothesized that the higher the women would score on the screening questions, the more likely they were to have high CB-PTSS or probable CB-PTSD scores or to meet the cut-off for both diagnoses. Corrections for multiple testing, such as Bonferroni corrections, were not used in this dataset, as we did not conduct the same test multiple times on a given hypothesis, rather, we had a high number of different covariates for which singular tests were conducted.

## 3 Results

**Table 1** shows detailed descriptive information regarding sociodemographic, medical, and mental health characteristics. The *life threat* and *infant threat* continuous scores were lower in the *community sample* than in the *ECS sample*. Thus, a post-hoc chi-square test was performed to examine the relation between the groups and the response to the *life threat* (i.e., *life threat* ≥ 3) and to the *infant threat* (i.e., *infant threat* ≥ 3). The relation between these variables was significant, X^2^ (1) = 18.24, p <0.001 and X^2^ (1) = 9.26, p = 0.002 for *life threat* and *infant threat* respectively. Thus, women belonging to the ECS sample were more likely to experience a perception of *life threat* or of *infant threat* than women belonging to the community sample.

### 3.1 Prospective associations between *life threat* or *infant threat* and CB-PTSS/PTSD at four to six weeks and six months

In the community sample, **Table 2** shows that the *life threat* continuous score was positively and significantly associated with PDS-F total score, and with the PDS-F avoidance score both at one month. In model 2, these associations disappeared in model 2 for the PDS-F total score (*β*: 0.93, *CI*: (-1.83, 3.70), *p*=0.25) and avoidance score at one month (*β*: 0.74, *CI*: (-0.64, 2.11), *p*=0.14). At six months, the *life threat* continuous score was associated with the PDS-F hyperarousal score. This was maintained (*β*: 0.98, *CI*: (0.43, 1.53), p<0.01) in model 2. The *infant threat* continuous score was positively and significantly associated with the PDS-F total score, the cut-off for meeting CB-PTSD, the avoidance and the hyperarousal scores at one month. In model 2, the *infant threat* continuous score was still positively and significantly associated with the PDS-F total score (*β*: 2.46, *CI*: (0.93, 4.00), *p*<0.01), the cut-off for meeting CB-PTSD (*β*: 1.10, *CI*: (0.38, 2.38), *p*=0.01), and the avoidance scores (*β*: 1.23, *CI*: (0.44, 2.02), *p*<0.01) at one month. Additionally, the *infant threat* continuous score was positively and significantly associated with the PDS-F total score (*β*: 0.70, *CI*: (-0.14, 1.53), *p*=0.05), the intrusions (*β*: 0.21, *CI*: (0.00, 0.41), *p*=0.03), and the hyperarousal (*β*: 0.50, *CI*: (-0.01, 1.02), *p*=0.03) scores only in model 2, at six months. In the ECS sample, **Table 2** shows there were no significant prospective associations between the *life threat* continuous score and CB-PTSD measures at six weeks or six months, even with covariates. The *infant threat* continuous score was positively and significantly associated with the PCL-5 total and cut-off scores, as well as with the PCL-5 intrusions and negative alteration cognitions and mood scores at six weeks. In model 2, the *infant threat* continuous score remained positively and significantly associated with the cut-off scores (*β*: 0.53, *CI*: (0.14, 1.03), *p*=0.01), with the PCL-5 intrusions (*β*: 0.39, *CI*: (-0.04, 0.82), *p*=0.04), and negative cognitions and mood (*β*: 0.43, *CI*: (-0.09, 0.95), *p*=0.05) scores at six weeks. The significant and positive association between the *infant threat* continuous score and the PCL-5 total at six weeks disappeared. At six months, the *infant threat* continuous score was prospectively associated with the PCL-5 total score and the PCL-5 intrusions, negative alterations of cognitions and mood, and the hyperarousal scores. In model 2, the *infant threat* continuous score was still positively and significantly associated with the PCL-5 total score (*β*: 1.85, *CI*: (-0.11, 3.81), *p*=0.03) and the PCL-5 intrusions (*β*: 0.38, *CI*: (-0.08, 0.84), *p*=0.05), negative alterations of cognitions and mood (*β*: 0.81, *CI*: (0.00, 1.62), *p*=0.03), and the hyperarousal (*β*: 0.60, *CI*: (-0.10, 1.30), *p*=0.05) scores at six months.

**Table 2:** Prospective associations between life threat and infant threat scores and maternal CB-PTSS.

Dependent variables	Life threat score	Infant threat score
Community sample		
	*N, β [95% CI], p*	*N, β [95% CI], p*
CB-PTSS Total score (one month) (PDS-F)	**53, 1.54 [-0.20; 3.27], 0.04**	**53, 1.4 [0.45, 2.35], 0.00**
Meeting the cut-off for CB-PTSD (one month) (PDS-F)	53, 0.45 [-0.34; 1.11], 0.09	**53, 0.69 [0.18, 1.35], 0.01**
CB-PTSS Intrusions (one month) (PDS-F)	53, 0.16 [-0.28; 0.61], 0.23	53, 0.16 [-0.11, 0.4], 0.13
CB-PTSS Avoidance (one month) (PDS-F)	**53, 0.87 [-0.02; 1.76], 0.03**	**53, 0.66 [0.17, 1.16], 0.01**
CB-PTSS Hyperarousal (one month) (PDS-F)	53, 0.50 [-0.18; 1.19], 0.08	**53, 0.58 [0.22, 0.95], 0.00**
CB-PTSS Total score (six months) (PDS-F)	44, 0.54 [-0.42; 1.49], 0.13	44, 0.31 [-0.29, 0.91], 0.15
Meeting the cut-off for CB-PTSD (six months) (PDS-F)	-	-
CB-PTSS Intrusions (six months) (PDS-F)	44, -0.03 [-0.29; 0.23], 0.42	44, 0.11 [-0.05, 0.27], 0.09
CB-PTSS Avoidance (six months) (PDS-F)	44, -0.10 [-0.52; 0.33], 0.32	44, -0.04 [-0.30, 0.23], 0.39
CB-PTSS Hyperarousal (six months) (PDS-F)	**44, 0.66 [0.10; 1.22], 0.01**	44, 0.24 [-0.13, 0.60], 0.10
ECS sample		
CB-PTSS Total score (six weeks) (PCL-5)	57, -0.08 [-1.54; 1.39], 0.54	**57, 1.24 [-0.06, 2.53], 0.03**
Meeting the cut-off for CB-PTSD (six weeks) (PCL-5)	57, 0.01 [-0.35; 0.33], 0.48	**57, 0.51, [0.16, 0.95], 0.00**
CB-PTSS Intrusions (six weeks) (PCL-5)	57, -0.03 [-0.45; 0.39], 0.45	**57, 0.38 [0.01, 0.74], 0.03**
CB-PTSS Avoidance (six weeks) (PCL-5)	57, 0.02 [-0.21; 0.25], 0.45	57, 0.1 [-0.11, 0.30], 0.18
CB-PTSS Negative alterations of cognitions and mood (six weeks) (PCL-5)	57, 0.22 [-0.28, 0.72], 0.45	**57, 0.4 [-0.04, 0.85], 0.04**
CB-PTSS Hyperarousal (six weeks) (PCL-5)	57, -0.28 [-0.85; 0.28], 0.16	57, 0.36 [-0.15, 0.88], 0.08
CB-PTSS Total score (six months) (PCL-5)	49, -0.65 [-2.47; 1.17], 0.24	**49, 1.67 [0.10, 3.24], 0.02**
Meeting the cut-off for CB-PTSD (six months) (PCL-5)	49, -0.13 [-0.55; 0.22], 0.25	49, 0.19 [-0.13, 0.54], 0.13
CB-PTSS Intrusions (six months) (PCL-5)	49, -0.03 [-0.44, 0.33], 0.45	**49, 0.38 [-0.02, 0.77], 0.03**
CB-PTSS Avoidance (six months) (PCL-5)	49, -0.06 [-0.31; 0.19], 0.32	49, 0.11 [-0.11, 0.33], 0.16
CB-PTSS Negative alterations of cognitions and mood (six months) (PCL-5)	49, -0.08 [-0.46, 0.26], 0.39	**49, 0.59 [-0.07, 1.25], 0.04**
CB-PTSS Hyperarousal (six months) (PCL-5)	49, 0.10 [-0.24; 0.43], 0.26	**49, 0.59 [0.03, 1.15], 0.02**

CB-PTSS: childbirth-related posttraumatic stress symptoms.

PDS-F: French version of the Post-traumatic Diagnostic Scale (range: 0−51).

PCL-5: Post-Traumatic Stress Disorder Checklist for Diagnostic and Statistical Manual of Mental Disorders, fifth edition (range: 0−80).

Results reported as β-Coefficient (95% confidence interval) from linear and logistic regressions.

Statistical significance level was defined as p <0.05.

The values in bold represent significant associations (i.e. p<0.05).

## 4 Discussion

In the community sample, eight percent of mothers perceived a threat to their life during childbirth *vs*. 40% to their infant’s life. In the ECS sample, 40% of mothers perceived a threat to their life *vs*. 66.7% to the infant’s. The fact that mothers were up to five times more likely to perceive their infant’s life to be threatened than their own is novel. Some authors have highlighted that the nature of the *life threat* is often misunderstood, particularly in the context of childbirth. Many studies assessing the traumatic stressor criterion assess both types of threat together, i.e., “Did you think you or your infant’s life was at risk?” (39).

The prevalence of probable self-reported CB-PTSD diagnosis decreased from 5.56% at one month to 0% at six months in the community sample, contrasting with prior findings (4). In the ECS sample, the prevalence rate of probable CB-PTSD increased from 2.67% at six weeks to 6.67% at six months. This is in line with a recent meta-analysis showing an increase between three to four and six months postpartum (13).

In both samples, both types of perceived threat during childbirth were important in predicting maternal CB-PTSS, particularly at four to six weeks postpartum. This is in line with a previous study showing that both types of perceived threat were reported to be higher in women with full CB-PTSD compared to those being non-symptomatic (40).

In the community sample, perceiving any threat during childbirth was prospectively associated with total scores of CB-PTSS and with several CB-PTSD subscales scores. However, in the ECS sample, *infant threat* was prospectively associated with CB-PTSD outcomes, whereas the *life threat* yielded no significant association with future CB-PTSS. It is possible that, in this sample, where the *objective* threat to the infant’s life was increased (most ECS are often a consequence of a threat to the infant’s health (41)), the probability of *subjectively* perceiving a threat to the infant’s life is also increased (42).

When looking at the CB-PTSD symptom clusters, both types of perceived threat were prospectively associated with hyperarousal, at one or six months, and with avoidance at four weeks in our community sample. In the ECS sample, there were no such similarities: the *infant threat* score was prospectively associated with most PTSD subscales but *not* avoidance. One explanation may be that different coping mechanisms are at play in this population, depending on the nature of the perceived threat. If mothers perceived their own life to be at risk during childbirth, avoiding any reminders may be a way of coping. If the infant’s life was perceived to be threatened, mothers may instead cope by being overly alert for signals indicating additional risks for their infant and be influenced by societal expectations and the perception of the maternal role and responsibility to protect infants from harm (43). Indeed, in this ECS sample, a prospective association with hyperarousal at six months was shown for the *infant threat* but not for the *life threat*. *Infant threat* was prospectively associated with intrusion symptoms at both four to six weeks and six months. When using the *infant threat*, but not the *life threat* continuous scores. It is thus possible that having feared for the infant during childbirth and subsequently being overly alert to further signals of *infant threat* may trigger more intrusions symptoms.

The role of covariates is also worth mentioning. In the community sample, the predictive power of *life threat* decreased when controlling for maternal age and mode of childbirth. This is not surprising, as the mode of childbirth is associated with *life threat* (44). However, the opposite effect was found when focusing on *infant threat* whilst controlling for Apgar score and birth weight: more CB-PTSD symptom clusters were prospectively associated with *infant threat*, both at four to six weeks, as well as at six months.

The strengths of this study include the use of a community as well as an ECS sample and the use of validated questionnaires to measure maternal CB-PTSS and probable CB-PTSD. At the time of data collection, no validated questionnaire to measure CB-PTSS or CB-PTSD existed; therefore, validated generic questionnaires assessing PTSD symptoms were used and their instructions adapted to assess childbirth-related symptoms.

Limitations are the use of different time points to assess *life threat* and *infant threat* and the use of different questionnaires to measure CB-PTSS and probable CB-PTSD in mothers across the two samples; thus, conclusions need to be cautiously drawn. More specifically, contrarily to our ECS sample for whom CB-PTSS and probable CB-PTSD were evaluated with the PCL-5, the PDS-F used in our community sample lacks an evaluation of negative changes in mood and cognition, which may be viewed as a limitation in our manuscript. Furthermore, in the covariates, there was missing data, due to the fact that women’s hospital records could not always be accessed. This means that regressions conducted with covariates should be interpreted with caution.

## 5 Conclusion

Our results have potential clinical implications: they show that assessing *life threat* and *infant threat* in mothers shortly after childbirth via two brief items may help to identify mothers at risk of developing CB-PTSS and probable CB-PTSD up to six months. This study may thus represent the first step in the development of a brief screening tool for CB-PTSS and probable CB-PTSD, which is needed in order to identify those at risk early and to prevent negative consequences for the family (39, 45). Future studies should replicate these results and assess emotional responses to threat, the psychometric properties as well as the acceptability of a brief screening tool.

## Data availability statement

Not all participants agreed to share their data with persons outside of the START Research Consortium. The ethical study protocol, including the statistical analysis plan, statistical code, and data dictionary are available upon request from the corresponding author.

## Ethics statement

The studies involving humans were approved by Ethics committee for research in humans of the Canton de Vaud. The studies were conducted in accordance with the local legislation and institutional requirements. The participants provided their written informed consent to participate in this study.

## Author contributions

LG: Conceptualization, Data curation, Investigation, Methodology, Writing – original draft, Writing – review & editing, Formal Analysis. VS: Conceptualization, Data curation, Investigation, Methodology, Writing – original draft, Writing – review & editing, Project administration. CD: Conceptualization, Data curation, Investigation, Methodology, Project administration, Writing – original draft, Writing – review & editing. AH: Conceptualization, Data curation, Investigation, Methodology, Project administration, Writing – original draft, Writing – review & editing, Funding acquisition, Resources, Supervision, Validation.
