# Supplementary material for: A two-item screening of maternal or infant perceived life threat during childbirth prospectively associated with childbirth-related posttraumatic stress symptoms up to six months postpartum: two observational longitudinal studies
Source: Front Psychiatry. 2024 Apr 9;15:1360189. doi: 10.3389/fpsyt.2024.1360189 (PMC11036539; doi:10.3389/fpsyt.2024.1360189)
Supplement: Supplementary file 1 [file Table_1.pdf]

Supplementary table. Prospective associations between maternal perceived life and infant threat scores and maternal CB-PTSD and CB-PTSS with covariates

**Dependent variables**	**Life threat score**	**Infant threat score**
**Community sample**	*Covariate: maternal age and mode of delivery*	*Covariates: Apgar score at five minutes and birth weight*
	N, β [95% CI], p	*N, β [95% CI], p*
CB-PTSS Total score (one month) (PDS-F)	34, 0.93 [-1.83, 3.70], 0.25	**34, 2.46 [0.93, 4.00], 0.00**
Meeting the cut-off for CB-PTSD (one month) (PDS-F)	34, -0.74 [-2.22, 0.34], 0.11	**34, 1.1 [0.38, 2.38], 0.01**
CB-PTSS Intrusions (one month) (PDS-F)	34, 0.20 [-0.51, 0.91], 0.57	34, 0.25 [-0.17, 0.67], 0.12
CB-PTSS Avoidance (one month) (PDS-F)	34, 0.74 [-0.64, 2.11], 0.14	**34, 1.23 [0.44, 2.02], 0.00**
CB-PTSS Hyperarousal (one month) (PDS-F)	34, 0.00 [-0.96, 0.96], 0.50	**34, 0.98 [0.43, 1.53], 0.00**
CB-PTSS Total score (six months) (PDS-F)	30, 0.80 [-0.61, 2.21], 0.13	**30, 0.70 [-0.14, 1.53], 0.05**
Meeting the cut-off for CB-PTSD (six months) (PDS-F)	-	-
CB-PTSS Intrusions (six months) (PDS-F)	30, -0.01 [-0.35, 0.33], 0.48	**30, 0.21 [0.00, 0.41], 0.03**
CB-PTSS Avoidance (six months) (PDS-F)	30, 0.11 [-0.48, 0.70], 0.36	30, -0.01 [-0.38, 0.35], 0.47
CB-PTSS Hyperarousal (six months) (PDS-F)	**30, 0.70 [-0.14, 1.54], 0.05**	**30, 0.50 [-0.01, 1.02], 0.03**
**ECS sample**	*Covariate: maternal age*	*Covariates: Apgar score at five minutes and birth weight*
CB-PTSS Total score (six weeks) (PCL-5)	46, -0.69 [-2.34, 0.95], 0.20	47, 1.23 [-0.29, 2.75], 0.06
Meeting the cut-off for CB-PTSD (six weeks) (PCL-5)	46, -0.12 [-0.58, 0.25], 0.27	**47, 0.53 [0.14, 1.03], 0.01**
CB-PTSS Intrusions (six weeks)	46, -0.16 [-0.65, 0.32], 0.25	**47, 0.39 [-0.04, 0.82], 0.04**
CB-PTSS Avoidance (six weeks)	46, -0.14 [-0.38, 0.10], 0.12	47, 0.07 [-0.15, 0.30], 0.26
CB-PTSS Negative alterations of cognitions and mood (six weeks)	46, 0.08 [-0.48, 0.65], 0.38	**47, 0.43 [-0.09, 0.95], 0.05**
CB-PTSS Hyperarousal (six weeks)	46, -0.47 [-1.11, 0.17], 0.08	47, 0.34 [-0.27, 0.96], 0.14
CB-PTSS Total score (six months) (PCL-5)	38, -1.05 [-3.24, 1.14], 0.17	**39, 1.85 [-0.11, 3.81], 0.03**
Meeting the cut-off for CB-PTSD (six months) (PCL-5)	38, -0.15 [-0.65, 0.25], 0.25	39, 0.26 [-0.11, 0.69], 0.10
CB-PTSS Intrusions (six months) (PCL-5)	38, -0.13 [-0.66, 0.39], 0.31	**39, 0.38 [-0.08, 0.84], 0.05**
CB-PTSS Avoidance (six months) (PCL-5)	38, -0.11 [-0.38, 0.16], 0.20	39, 0.06 [-0.20, 0.33], 0.31
CB-PTSS Negative alterations of cognitions and mood (six months) (PCL-5)	38, -0.58 [-1.49, 0.34], 0.11	**39, 0.81 [0.00, 1.62], 0.03**
CB-PTSS Hyperarousal (six months) (PCL-5)	38, -0.23 [-1.0, 0.54], 0.28	**39, 0.6 [-0.10, 1.29], 0.05**

CB-PTSS: childbirth-related posttraumatic stress symptoms
PDS-F: French version of the Post-traumatic Diagnostic Scale (range: 0−51)
PCL-5: Post-Traumatic Stress Disorder Checklist for Diagnostic and Statistical Manual of Mental Disorders, fifth edition (range: 0−80)
PTSD: Post-Traumatic Stress Disorder
Results reported as β-Coefficient (95% confidence interval) from linear and logistic regressions.
Statistical significance level was defined as p <0.05 and presented in bold.
